# Supplementary material for: Upregulation of HPV16E1 and E7 expression and FOXO3a mRNA downregulation in high-grade cervical neoplasia
Source: PeerJ. 2024 Dec 6;12:e18601. doi: 10.7717/peerj.18601 (PMC11627083; doi:10.7717/peerj.18601)
Supplement: Table S1 [file peerj-12-18601-s001.pdf]

Table S1. The specific primers

Target		Sequences (5' to 3')	Size	References
HPV16E1	F	GCGGGTATGGCAATACTGAA	147 bp	Bogovac et al. 2011
	R	TAACACCCTCTCCCCCACTT		
HPVE4	F	AAGCCGTSKCCKTGGGCAC	191 bp	Supchokpul et al. 2011
	R	TATRGGYGTAGTGTTACYAYTACAG		
HPV16E6	F	CGACGTGAGGTATATGACTTTGC	222 bp	Baedyananda et al. 2017
	R	AGGACACAGGACACAGTGGCTTTTGACA		
HPV16E6*I	F	ACTGCGACGTGAGGTGTATTAAC	83 bp	Chaiwongkot et al. 2020
	R	TGGAATCTTTGCTTTTTGTCC		
HPV16E7 FL	F	CAGCTCAGAGGAGGAGGATG	166 bp	Chaiwongkot et al. 2020
	R	GCCCATTAACAGGTCTTCCA		
FOXO3a mRNA	F	TTCAAGGATAAGGGCGACAGCAAC	302 bp	Kannike et al. 2014
	R	CTGCCAGGCCACTTGGAGAG		
GAPDH mRNA	F	GAGTCAACGGATTTGGTCGT	238 bp	Joseph et al. 2012
	R	TTGATTTTGGAGGGATCTCG		

Degenerate Bases: R=A or G; Y=C or T; K=G or T; S=G or C

All primers were purchased from Macrogen (Korea).
