# Supplementary material for: Upregulation of HPV16E1 and E7 expression and FOXO3a mRNA downregulation in high-grade cervical neoplasia
Source: PeerJ. 2024 Dec 6;12:e18601. doi: 10.7717/peerj.18601 (PMC11627083; doi:10.7717/peerj.18601)
Supplement: Table S2 [file peerj-12-18601-s002.pdf]

Table S2. Age distribution among the groups based on cytology results

Target	Cytology	Age		P-value (One-way ANOVA)
		Mean	SEM	
HPV16E1	NILM	42.43	2.13	0.7638
	ASC-US	38.42	2.33	
	LSIL	40.88	4.31	
	ASC-H	42.25	2.69	
	HSIL	44.13	7.30	
HPVE4	NILM	42.05	3.05	0.8629
	ASC-US	38.95	2.73	
	LSIL	40.88	4.31	
	ASC-H	44.67	1.67	
	HSIL	44.29	8.43	
HPV16E6	NILM	42.65	2.21	0.7936
	ASC-US	38.75	2.35	
	LSIL	40.89	4.31	
	ASC-H	42.25	2.69	
	HSIL	44.13	7.30	
HPV16E6*I	NILM	43.82	2.54	0.8011
	ASC-US	39.52	2.59	
	LSIL	41.14	4.96	
	ASC-H	45.75	1.60	
	HSIL	44.29	8.43	
HPV16E7	NILM	42.69	2.15	0.6902
	ASC-US	38.68	2.25	
	LSIL	40.88	4.31	
	ASC-H	45.75	1.60	
	HSIL	44.13	7.30	
FOXO3a	NILM	42.57	2.11	0.6537
	ASC-US	38.48	2.43	
	LSIL	38.83	5.20	
	ASC-H	45.75	1.60	
	HSIL	44.13	7.30	
