# Supplementary figures and images for: Upregulation of HPV16E1 and E7 expression and FOXO3a mRNA downregulation in high-grade cervical neoplasia
Source: PeerJ. 2024 Dec 6;12:e18601. doi: 10.7717/peerj.18601 (PMC11627083; doi:10.7717/peerj.18601)

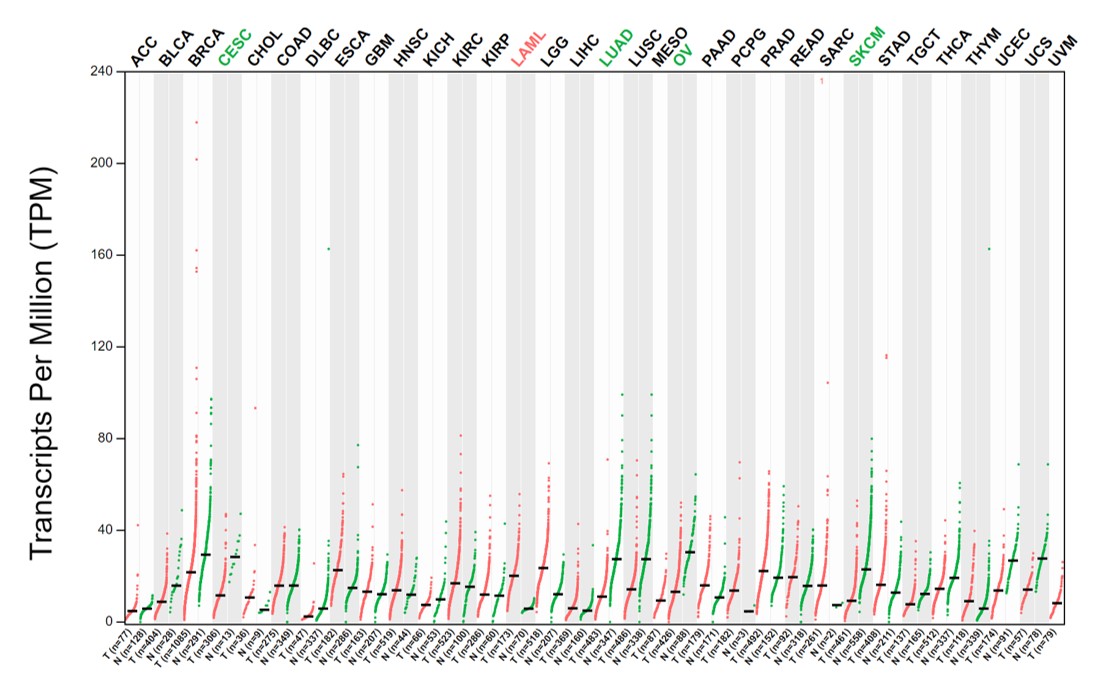

Supplement: Figure S1 — Each dot represents an expression of samples. Y axis, expression level of FOXO3a based on the RNA-seq data. X axis, different types of cancers and matched normal data. Green text = downregulate in cancer; red text = upregulate in cancer. (Abbreviation: ACC = Adrenocortical carcinoma; BLCA = Bladder Urothelial Carcinoma; BRCA = Breast invasive carcinoma; CESC = Cervical squamous cell carcinoma and endocervical adenocarcinoma; CHOL = Cholangio carcinoma; COAD = Colon adenocarcinoma; DLBC = Lymphoid Neoplasm Diffuse Large B-cell Lymphoma; ESCA = Esophageal carcinoma; GBM = Glioblastoma multiforme; HNSC = Head and Neck squamous cell carcinoma; KICH = Kidney Chromophobe; KIRC = Kidney renal clear cell carcinoma; KIRP = Kidney renal papillary cell carcinoma; LAML = Acute Myeloid Leukemia; LGG = Brain Lower Grade Glioma; LIHC = Liver hepatocellular carcinoma; LUAD = Lung adenocarcinoma; LUSC = Lung squamous cell carcinoma; MESO = Mesothelioma; OV = Ovarian serous cystadenocarcinoma; PAAD= Pancreatic adenocarcinoma; PCPG = Pheochromocytoma and Paraganglioma; PRAD =Prostate adenocarcinoma; READ = Rectum adenocarcinoma; SARC = Sarcoma; SKCM =Skin Cutaneous Melanoma; STAD = Stomach adenocarcinoma; TGCT = Testicular Germ Cell Tumors; THCA = Thyroid carcinoma; THYM = Thymoma; UCEC = Uterine Corpus Endometrial Carcinoma; UCS = Uterine Carcinosarcoma; UVM = Uveal Melanoma) [file peerj-12-18601-s003.jpg]

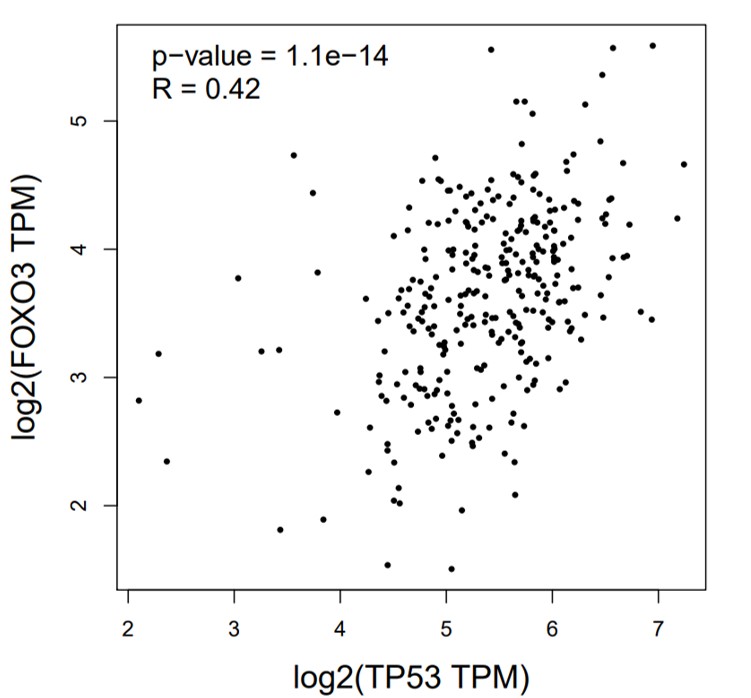

Supplement: Figure S2 — The datasets used for the analyses were obtained from The Cancer Genome Atlas (TCGA) via the GEPIA2 web interface. [file peerj-12-18601-s004.jpg]

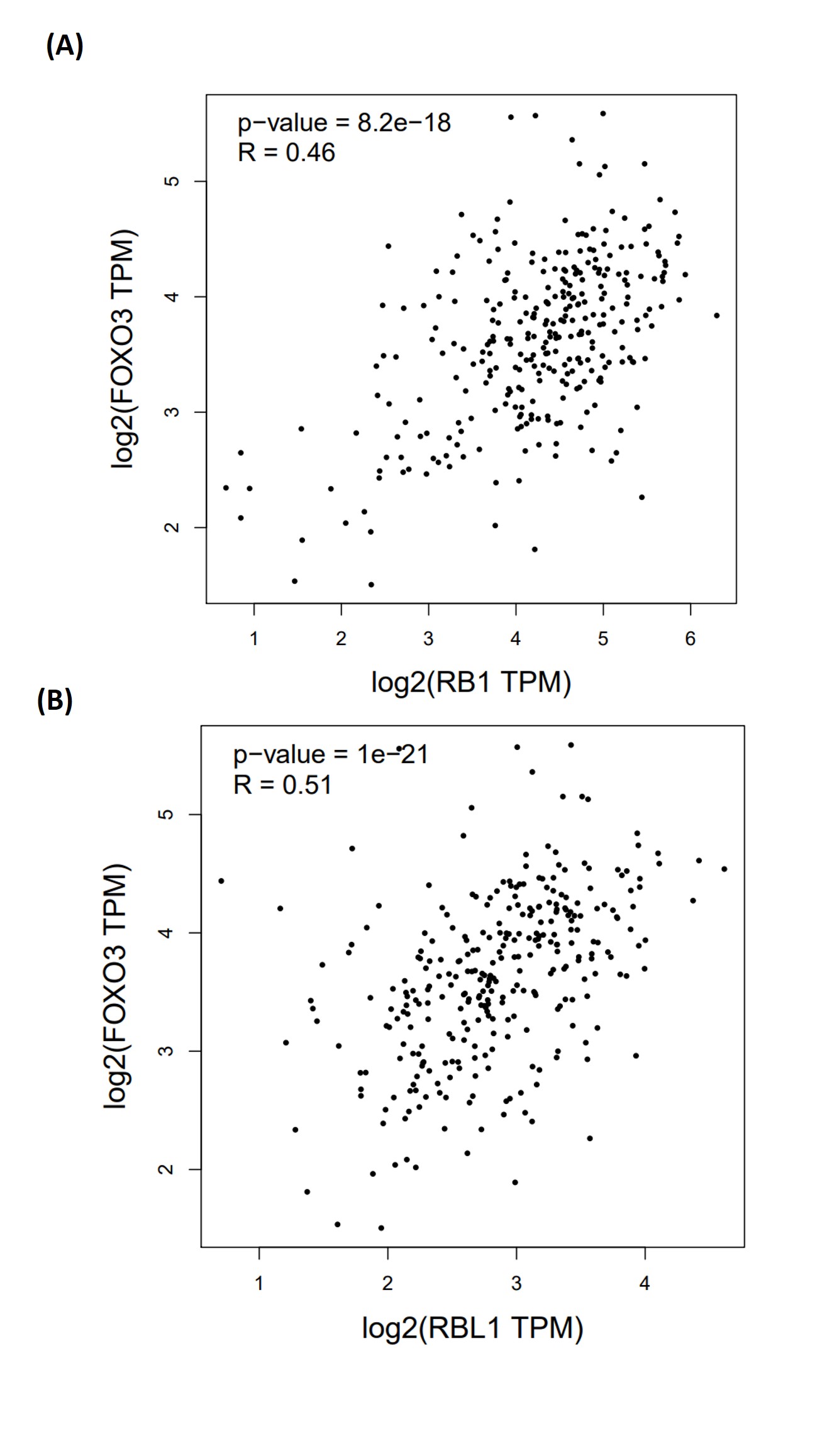

Supplement: Figure S3 — The datasets used for the analyses were obtained from The Cancer Genome Atlas (TCGA) via the GEPIA2 web interface. [file peerj-12-18601-s005.jpg]
